# LPS-induced inflammation differentially affects endogenous Ca^2^⁺ activity in mouse and human iPSC-derived astrocytes

**DOI:** 10.1186/s10020-026-01450-3

**Published:** 2026-03-05

**Authors:** Franziska E. Müller, Flavian Ivanov, Anne-Catharine Studt, Ida Nitzsche, Frauke S. Bahr, Anna-Lena Krüger, Josephine Labus, Ghanendra Singh, Evgeni G. Ponimaskin, Kerstin Lenk, Andre Zeug

**Affiliations:** 1 Cellular Neurophysiology, Institute of Neurophysiology, Hannover Medical School, Hannover, Germany; 2 Institute of Neural Engineering, Graz University of Technology, Graz, Austria

**Keywords:** Astrocytes, Lipopolysaccharide, Ca^2+^ signalling, Calcium, Heterogeneity

## Abstract

**Background:**

Mouse and human astrocytes exhibit substantial species-specific differences in both morphology and function. Their response to inflammatory stimuli, however, remains underexplored despite being crucial for understanding bidirectional astrocyte-neuron signaling dynamics and for translating preclinical findings to human-relevant applications. Induced pluripotent stem cell-based models thus offer a powerful platform to investigate these mechanisms in the context of the human neural connectome.

**Methods:**

We apply two well-established in vitro protocols by exposing cultured astrocytes to lipopolysaccharide (LPS) for either 3 or 24 h to trigger an inflammatory response. We investigated how LPS-induced inflammation affects the endogenous Ca^2+^ activity in astrocytes derived from the mouse hippocampus (HC) and prefrontal cortex (PFC), as well as human induced pluripotent stem cell (hiPSC)-derived astrocytes. Both, morphological changes and Ca^2+^ activity were analyzed using the volume fraction (VF) approach and our previously developed multi-threshold event detection (MTED) combined with machine learning-driven non-negative matrix factorization (NMF).

**Results:**

The comprehensive assessment of Ca^2+^ activity patterns and their relation to cell morphology revealed significant alterations in response to LPS treatment, and further between mouse and human hiPSC-derived astrocytes. While both mouse and human astrocytes show increased Ca^2+^ event frequency after short-term LPS exposure, after 24 h of LPS treatment Ca^2+^ activity is severely restricted in PFC astrocytes but substantially increased in human astrocytes.

**Conclusions:**

Our findings highlight the unique properties of human iPSC-derived astrocytes and provide detailed insights into how Ca^2+^ signaling becomes dysregulated under neuroinflammatory conditions. Understanding the species-specific responses is essential for advancing stem cell-based models of human astrocyte-neuron signaling circuits and for developing targeted therapeutic strategies to alleviate neuroinflammation and Ca^2+^-related dysregulation in neurological diseases.

**Supplementary Information:**

The online version contains supplementary material available at 10.1186/s10020-026-01450-3.

## Background

Inflammation is the common hallmark of neurodegenerative diseases, such as Alzheimer’s disease (AD), Frontotemporal dementia (FTD), Parkinson’s disease (PD), Amyotrophic lateral sclerosis (ALS) and Huntington’s disease (HD). It is suspected, that chronic inflammation in the central nervous system (CNS) is not simply a consequence of these diseases, but could be a driver, and even an initiator (Zhang et al. 2023). Neuroinflammation is fundamentally driven by glial cells in the brain, including astrocytes and microglia. Astrocytes are considered promising therapeutic targets due to their dual role in both exacerbating and mitigating neuroinflammation (González-Reyes et al. 2017; Dittlau and Freude 2024; Zhao et al. 2024). They further exhibit remarkable heterogeneity within and between different brain regions, and were therefore recently classified into developmentally- or stimulus-induced astrocytes. Identifying these subsets of astrocytes is crucial in developing astrocyte-targeted drugs for neurodegenerative diseases (Lee et al. 2022; Endo et al. 2022).

Astrocytes respond to inflammation by acquiring a reactive phenotype, which is characterized by changes in their molecular signature, morphology and functions (Hasel et al. 2021; Patani et al. 2023). Initially, astrocytes were divided into A1 pro-inflammatory reactive astrocytes and A2 astrocytes with an anti-inflammatory signature (Liddelow et al. 2017). However, the last years have questioned this bipolar division and it is suggested that astrocyte heterogeneity is much more complex. Whether astrocyte heterogeneity in the healthy brain influences astrocyte reactivity upon pathology is still an open question (Liddelow and, Barres 2017; Escartin et al. 2021).

One of the unique functional features of astrocytes are intracellular fluctuations of calcium (Ca^2+^), which are regarded as a special form of signalling, governing gliotransmitter release and maintaining neuronal homeostasis (Verkhratsky et al., 1998; Verkhratsky and Nedergaard 2018; Semyanov et al. 2020). These Ca^2+^ dynamics are highly complex, as frequency, amplitude and spatial extent may all encode information and determine or modulate downstream signalling (Shigetomi et al. 2019). This is particularly critical at the tripartite synapse, where astrocyte Ca^2+^ activity modulates neuronal excitability and shapes neuronal network dynamics (Benedetti et al. 2011). Relatedly, astrocyte Ca^2+^ signalling is disturbed in many neurodegenerative diseases (Shigetomi et al. 2019), as shown for AD (Kuchibhotla et al. 2009; Vincent et al. 2010; Shah et al. 2022), PD (Ramos-Gonzalez et al. 2021; Bancroft and Srinivasan 2022), ALS (Martorana et al. 2012; Kawamata et al. 2014; Yang et al. 2024) and HD (Jiang et al. 2016; Barry et al. 2022). Studying these processes in human induced pluripotent stem cell (hiPSC)-derived models offers a valuable opportunity to dissect astrocyte-neuron interactions and their involvement in circuit-level dysfunction in disease.

Primary cultures of mouse astrocytes have been widely accepted as a valuable model to study astrocyte function (Lange et al. 2012). In addition, the possibility of differentiating human pluripotent stem cells into astrocytes provides an intriguing opportunity to investigate the human counterpart (Krencik et al. 2011; Lanciotti et al. 2021). Mouse and human astrocytes exhibit common, but also species-specific properties, which are reflected in their size and complexity (Oberheim et al. 2009), their physiological functions in the brain (Verkhratsky et al. 2018), as well as their response to inflammation (Li et al. 2021). One widely-used model to induce inflammatory signalling in vivo and in vitro is the treatment with lipopolysaccharide (LPS). LPS is a component of the outer bacterial wall of gram-negative bacteria, and many cells, including astrocytes, express LPS receptors on the cell surface (Tarassishin et al. 2014). The effect of LPS treatment on astrocytic inflammatory features was partly investigated (Tarassishin et al. 2014; Zhang et al. 2016). However, it is still not fully elucidated how LPS affects the complex endogenous Ca^2+^ activity of mouse and human astrocytes.

In this study, we assess (i) how LPS treatment affects the endogenous Ca^2+^ activity of mouse astrocytes from different brain regions, the hippocampus (HC) and prefrontal cortex (PFC). We compare (ii) the response of mouse and human iPSC-derived astrocytes to LPS. Distinct patterns of Ca^2+^ activity following LPS-induced inflammation in mouse HC and PFC astrocytes were identified, indicating that specific brain regions might be particularly vulnerable to inflammatory signalling. We further recognized a distinctive response of human iPSC-derived astrocytes to LPS stimulation, challenging the translation of experimental data from mouse to human, which is critical to consider in the development of therapeutic strategies. Understanding the changes caused by inflammation is the foundation to intercept aberrant Ca^2+^ signalling in astrocytes and use this as an entry route to treat neuroinflammatory conditions.

## Methods

### Aim of the study

The aim of this study was to dissect and compare the Ca^2+^ activity in mouse and human astrocytes under basal conditions and in response to LPS-induced inflammatory signalling.

### Mouse primary astrocyte culture

Primary astrocyte cell cultures were prepared from the isolated hippocampus (HC) and prefrontal cortices (PFCs) from neonatal mice as previously described by Wu et al*.* (Wu et al. 2014), with slight modifications. Cells from dissociated HC or PFC were seeded at a density of 5 × 10^4^ cells per 12 mm glass coverslip for microscopy in 500 µl plating medium (49 ml Minimal Essential Medium, 1 ml B-27, 500 µl 200 mM glutamine, 500 µl 100 mM sodium pyruvate, 5 U/ml penicillin, 5 mg/ml streptomycin). The entire plating medium was replaced after 3 days with 1 ml maintenance medium (49 ml Neurobasal-A medium, 1 ml B-27, 500 µl 200 mM glutamine, 5 u/ml penicillin, 5 mg/ml streptomycin). On day in vitro (DIV)11, ½ of the medium was exchanged with fresh maintenance medium prior to infection of the cells with adeno-associated viruses. Astrocytes were used for experiments between DIV14-17. All cell cultures were maintained at 37 °C in a humidified incubator in a 5% CO_2_ atmosphere until they were used for experiments.

### Human iPSC-derived astrocyte differentiation

Human astrocytes were differentiated from induced pluripotent stem cell (iPSC)-derived neural stem cells (NSCs). NSCs acquired from Axol Bioscience (Cambridge, UK) were derived from fibroblasts of a 64-year-old female donor (ax0019).

Cells were seeded in 2 ml NSC Expansion medium (96 ml Knockout™ DMEM/F-12, 1 ml GlutaMax™-I supplement, 20 ng/ml hFGF2, 20 ng/ml hEGF, 2 ml StemPro Neural Supplement, 1 ml Penicillin/Streptamycin) in 3 wells of a 6 well plate until confluent. For differentiation, cells were seeded in NSC Expansion medium supplied with ROCK-inhibitor Y-27632 (1:1000, S6390, Selleckchem, Houston, TX) on prepared ready-to-use-Geltrex (A1569601, ThermoFisher Scientific, Waltham, MA) or diluted Geltrex (125 µl in 40 ml in PBS; A14133-02, ThermoFisher Scientific, Waltham, MA)-covered wells at a density of 1.0 × 10^5^ cells/well in 6-well plates, 1.5 × 10^5^ cells/well in 12 well plates and 7.5 × 10^4^ cells/well in 24 well plates. In 24 well plates, cells were seeded onto coated glass coverslips for microscopy. On day 1 after seeding for differentiation, cells were changed into Astrocyte Differentiation medium (DMEM High Glucose Media (41965062), GlutaMaxTM-I supplement (5050061), N-2 TM Supplement, (17502048), Pencillin/Streptamycin (15140122), Fetal bovine serum (1027–106), all ThermoFisher Scientific, Waltham, MA). Starting DIV30, astrocytes were changed to Astrocyte differentiation medium without fetal bovine serum. Medium was renewed three times per week. Astrocytes were used for experiments on DIV42-DIV68.

### LPS stimulation

Astrocytes were either stimulated with 10 µg/ml LPS (#L8274, Sigma Aldrich, St. Louis, USA) for 3 h or with 100 ng/ml LPS for 24 h, prepared from a 1 mg/ml LPS stock solution. Mouse primary astrocytes were stimulated starting DIV14, while hiPSC-derived astrocytes were stimulated starting DIV53.

### AAV-infection

Mouse primary astrocytes were infected on DIV11 with 1 × 10^6^ viral genomes of AAV-hGFAP-tdTomato-V5-GCaMP6s, linking the green Calcium-sensitive GCaMP6s with the bright fluorescent protein tdTomato via a V5 linker sequence, to provide 1:1 stoichiometry. This AAV enables parallel morphological and functional Ca^2+^-imaging of astrocytes in the same recording. Mouse astrocytes were recorded starting 3 days later, between DIV14-17. Human iPSC-derived astrocytes were infected on DIV46 before imaging started 7 days later.

### Functional imaging and quantitative analysis

Ca^2+^ imaging was conducted on an upright Andor Spinning Disk microscope (Oxford Instruments, Belfast, Northern Ireland) equipped with a CSU-X1 (Yokogawa, Musashino, Japan) using split filter cubes (LPXR560, ET525/50, LP561) for simultaneous imaging of GCaMP6s and tdTomato. Cells were recorded for 10 min with 5 frames/s using a single excitation wavelength of 488 nm, which has the advantage of homogeneous relative excitation of both fluorophores leading to comparable brightness in both channels. Spectral unmixing was applied to compensate for the spectral overlap of the fluorophores. During microscopy, cells were kept in a balanced salt solution containing 115 mM NaCl, 5.4 mM KCl, 1 mM MgCl_2_, 2 mM CaCl_2_, and 20 mM HEPES, adjusted to pH 7.4 and osmolarity to 270–380 mOsm with glucose, respective to the culture. Live-cell imaging was performed at 37 °C. The temperature during the measurement was controlled by a custom-built heating device, heating the objective, stage and chamber to achieve thermal stability and avoid artefacts. Ca^2+^ recordings were evaluated using the previously developed multi-threshold event detection (MTED) algorithm (Müller et al. 2021), which we adapted for the use of our ratiometric Ca^2+^ biosensor tdTomato-V5-GCaMP6s. In brief, the Ca^2+^ activity was evaluated as $\Delta F/{F}_{R}=\left(F-{F}_{R}\right)/{F}_{R}$, where $F$ is the GCaMP6s fluorescence signal and ${F}_{R}$ the scaled tdTomato fluorescence. The microscope system specific scaling parameter for ${F}_{R}$ was obtained by evaluating the ratio of ${F}_{0}/{F}_{R}$, where ${F}_{0}$ is the approximated minimal fluorescence for each pixel, which reflects GCaMP brightness at basal Ca^2+^ levels (Müller et al. 2021). The same ${F}_{0}$ calculation algorithm was applied to the tdTomato signal to correct for remaining perturbations after spectral unmixing, resulting in an ${F}_{R}$ with excellent signal-to-noise ratio suitable for morphological investigations. The scaling factor for ${F}_{R}$ is selected to reflect ${F}_{R}$ for the GCaMP6s fluorescence at zero Ca^2+^, using the GCaMP6s parameters (Kd = 144 nM, n = 2.45 (Dana et al. 2019)) and assuming an eligible basal Ca^2+^ concentration of ~ 50 nM in our astrocyte cultures. The same scaling factor was used for all measurements. $\Delta F/{F}_{R}$ was then used for our MTED algorithm to determine Ca^2+^ event characteristics at various $\Delta F/{F}_{R}$ levels, which we name Ca^2+^ thresholds. Statistics, such as maximum event size, event duration, event distance and maximum slope of Ca^2+^ change (see Suppl. Table T1) were accumulated over the recordings and visualized in 2D histograms as color-coded frequency distributions of the corresponding properties for various Ca^2+^ thresholds. Thereby, the detected Ca^2+^ events from each recording are scaled to the number of cells in the field of view. Ca^2+^ activity was analysed over the complete cell, regardless of the subcellular localization of the event.

Fixed astrocytes were imaged on a Zeiss LSM780 confocal microscope with a C-Apochromat 40x/1.2 W objective and Zen2013 imaging software in online-fingerprinting mode with previously defined spectra for each fluorescence component obtained from single staining.

### RT-qPCR

RNA was isolated from cultured HC and PFC astrocytes at DIV17 and hiPSC-derived astrocytes at DIV68 using the RNeasy Mini Plus Kit according to the manufacturer’s protocol (#74104, Qiagen, Hilden, Germany). Subsequently, mRNA was transcribed to cDNA using LunaScript™ RT SuperMix Kit (#E3010L, NEB, Frankfurt, Germany). The cDNA was diluted 1:10 in H_2_O, and RTqPCR was performed using Luna® Universal Probe qPCR Master Mix (#M3004E, NEB, Frankfurt, Germany) on a QuantStudio 6 Flex RTqPCR machine (ThermoFisher Scientific, Waltham, MA). Expression levels were detected using TaqMan Primers and Probes (ThermoFisher Scientific, Waltham, MA) for GFAP (Hs00909233_m1, Mm01253033_m1), CD14 (Hs02621496_s1, Mm01158466_g1), TLR4 (Hs00152939_m1, Mm00445273_m1), Aldh1L1 (Hs01003842_m1), Nestin (Hs04187831_g1), NeuN (Hs01370654_m1), Vimentin (Hs00958111_m1), Sox2 (Hs04234836_s1), S100b (Hs00902901_m1), IL-1b (Hs01555410_m1, Mm00434228_m1), IL-6 (Hs00174131_m1, Mm00446190_m1), TNF-α (Hs00174128_m1, Mm00443258_m1), Iba-1 (Mm00479862_g1), SLC1A3 (Hs00904823_m1) and SLC1A2 (Hs01102423_m1). Expression levels were normalized to the mean of house-keeping gene GAPDH, which was detected using the following primer mix: forward 5′-TGCACCACCAACTGCTTAGC-3′, reverse 5′-GGCATGGACTGTGGTCATGAG-3′, probe 5′−6-FAM-CCCTGGCCAAGGTCATCCATGACAAC-TAM-3′ (Sigma, St. Louis, MO) and beta actin (Hs99999903_m1, Mm02619580_g1).

### ELISA assay

In order to evaluate the secreted TNF-α levels in the supernatant of hiPSC-derived astrocytes, a human TNF-α Immunoassay (#DTA00C, R&D Systems, Minneapolis, MN) was performed in accordance with the manufacturer's protocol.

### Western blot

For Western blot analysis, mouse astrocytes were lysed on DIV17 and hiPSC-derived astrocytes on DIV68. After washing cells with cold PBS, cells were scraped-off in lysis buffer (20 mM HEPES, pH 7.4, 100 nM NaCl, 100 nM NaF, 1 mM sodium orthovanadate, 5 mM EDTA, 1% Triton X-100 and protease inhibitors CLAP and PMSF) and centrifuged at 18,000 g for 15 min at 4 °C. 6 × SDS loading dye containing 5% β-mercaptoethanol was added to the supernatants, and boiled at 95 °C for 5 min before direct loading on 10% SDS–polyacrylamide gels. After separation, proteins were transferred onto nitrocellulose membranes and blocked in 5% milk or 5% BSA in TBS-T (0.2 M Tris, 5 M NaCl, 0.1% Tween) for 1 h at room temperature. The membranes were incubated with the following antibodies (in 5% milk if not stated otherwise): GFAP (ab4674, Abcam, Cambridge, UK), CD14 (56082 T, CellSignaling, Danvers, MA), TLR4 (PA5-142481, ThermoFisher Scientific, Waltham, MA) in 5% BSA, Aldh1L1 (ab235197, Abcam, Cambridge, UK), actin (A2066, Sigma Aldrich, St. Louis, USA). Western blots were densitometrically quantified using a custom-written MATLAB script and normalized by the sum-of-the-replicates method (Degasperi et al. 2014).

### Immunocytochemistry

Immunocytochemistry was performed on mouse astrocyte cultures at DIV17 and hiPSC-derived astrocytes at DIV68. Cells were fixed with 4% PFA for 10 min. Mouse astrocytes were incubated in 0.2% Triton-X in 5% BSA (w/v; BSA, #8076.4, Carl Roth, Karlsruhe, Germany, in PBS) for 1 h at RT. Human astrocytes were permeabilized using 0.3% Triton-X in PBS for 10 min at RT followed by incubation in 5% normal donkey serum for 1 h at RT. Cells were subsequently incubated with primary antibodies (overnight) and secondary antibodies (1–2 h) at RT.

Following primary antibodies were used for immunocytochemistry: GFAP (ab4674, Abcam, Cambridge, UK), CD14 (MA5-32248, ThermoFisher Scientific, Waltham, MA), TLR4 (PA5-142481, ThermoFisher Scientific, Waltham, MA), Aldh1L1 (ab235197, Abcam, Cambridge, UK), Nestin (312 011, SynapticSystems, Göttingen, Germany), NeuN (abn78, Merck, Darmstadt, Germany), Vimentin (MA5-11883, ThermoFisher Scientific, Waltham, MA), Sox2 (3579S, CellSignaling, Danvers, MA) S100b (AMAB91038-25UL, Atlas Antibodies, Bromma, Sweden), Iba-1 (C290A, Biocare Medical, Concord, CA).

Following secondary antibodies were used: donkey anti-mouse Alexa Fluor 594 (A21203, ThermoFisher Scientific, Waltham, MA), donkey anti-rabbit Alexa Fluor 488 (ABN78, Merck, Darmstadt, Germany), donkey anti-chicken DyLight 647, donkey anti-goat Alexa Fluor 488, donkey anti-goat Alexa Fluor 594 (#703–605-155, #705–545-147, #705–585-147, Jackson ImmunoResearch, Ely, UK; all diluted 1:400 in PBS). Cells were mounted using Fluoromount G mounting medium (#0100–01 SouthernBiotech, Birmingham, USA) and used 48 h later for microscopy.

### Caspase assay

In order to evaluate apoptosis, a Caspase3/7 assay was performed in accordance with the manufacturers protocol. In brief, after 24 h of control or LPS-treatment (100 ng/ml) CellEvent Caspase-3/7 Detection Reagent (#C10432, Invitrogen, Carlsbad, CA) 100 × stock solution was diluted 1:10 in conditioned medium of the respective well. Astrocytes were stained for 50 min before Hoechst 33342 (1:2000, #H3570, Invitrogen, Carlsbad, CA) was added for 10 min to label the nuclei. After a total of 1 h incubation, the staining mixture was discarded and astrocytes were imaged using Zeiss AxioObserver.Z1/7 with Zen 3.2 Blue software (Carl Zeiss, Jena). Image analysis was conducted by applying a self-written FIJI-macro using particle analyzer plugin and the built-in measure function. The median intensity of the TexasRed staining was scaled to the number of nuclei in order to account for variability in cell density.

### Nuclei analysis

Size and perimeter of astrocytic nuclei were analysed with Fiji software using the ROI manager, blow/lasso and measure tools.

### LDA, component analysis and weight matrix generation

Linear Discriminant Analysis (LDA) is a supervised machine learning technique for classification and dimensionality reduction, which was applied to the HC, PFC, and hiPSC datasets separately. It identifies a linear combination of features that maximizes separation between classes — here Ctrl., 3 h LPS, and 24 h LPS treatment — while minimizing variance within each class.

Non-Negative Matrix Factorization (NMF) can be used for the decomposition for multivariate data. The unsupervised machine learning technique factorizes a non-negative matrix X into the product of two lower rank matrices W and H, with X≈WH, where W are the weights for the feature components H. We applied NMF to the combined feature set of MaxSize, duration, distance, and Ca^2+^ change.

The LDA and NMF, as part of the "sklearn" library, were used in a custom-written Python script. If not mentioned otherwise, default parameters were used.

### Statistical evaluation

GraphPad Prism version 10 (La Jolla, CA, USA) was used for graph generation and statistical analysis. Data are presented as mean and standard error of the mean (SEM), as indicated in the figure legends. Data were tested for Gaussian distribution using Shapiro–Wilk normality test. The mRNA expression levels of GFAP, CD14 and TLR4 were compared between HC and PFC astrocytes using paired t-test, and in hiPSC-derived astrocytes at various time points using Kruskal–Wallis test. Western Blot results of hiPSC-derived astrocytes without or with LPS stimulation were compared using paired t-test. Basal Ca^2+^ levels and cell nuclei sizes were compared using Kruskal–Wallis test, while cumulative distributions of Ca^2+^ event characteristics were analyzed using 2-way ANOVA followed by Tukey’s multiple comparisons posthoc test. The following p-values were considered to be statistically significant: * *p* < 0.05; ** *p* < 0.01; *** *p* < 0.001; **** *p* < 0.0001.

## Results

### Cultured mouse hippocampal and prefrontal cortex astrocytes express LPS receptors CD14 and TLR4

Astrocytes are known to exhibit very distinct morphologies and fulfill selective functions according to their location in the brain, and such differences are also preserved in primary cultures of astrocytes.

**Fig. 1:**
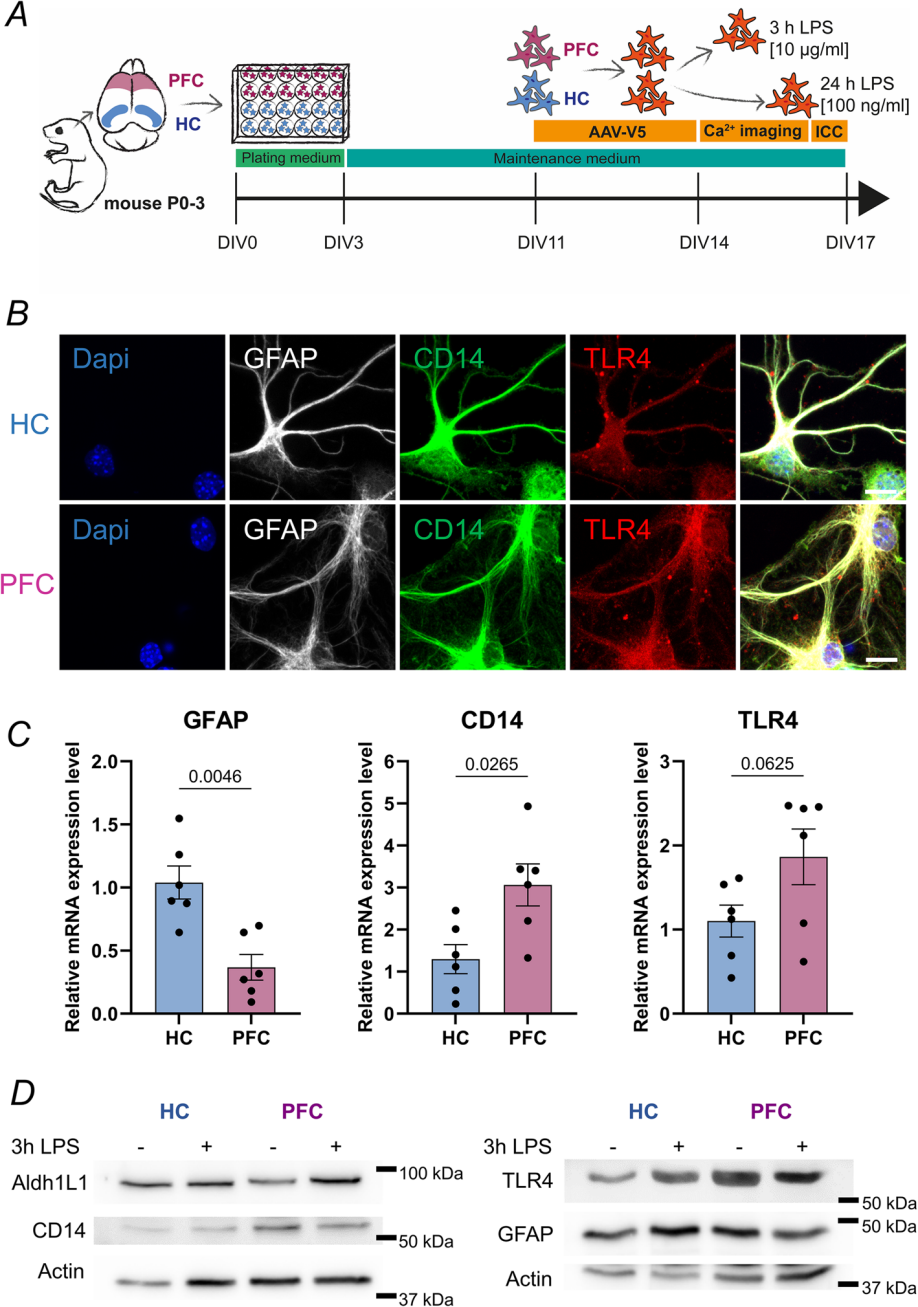
Mouse hippocampal and prefrontal cortex astrocytes express LPS receptors CD14 and TLR4 in vitro. **A** Scheme of experimental procedure. Brains of neonatal mice were dissected and hippocampal (HC) and prefrontal cortex (PFC) cells were seeded separately on a 24-well plate. At day in vitro (DIV)11 astrocytes were infected with AAV-hGFAP-tdTomato-V5-GCaMP6s (AAV-V5). Starting DIV14, astrocytes were either stimulated with 10 µg/ml LPS for 3 h or 100 ng/ml LPS for 24 h before recording their Ca^2+^ activity. Ca^2+^ imaging was conducted between DIV14-17, Immunocytochemistry (ICC) at DIV17. **B** Immunocytochemistry shows expression of GFAP, CD14 and TLR4 in mouse HC and PFC astrocytes at DIV17. Scale bars 10 µm. **C** Relative mRNA expression levels of GFAP, CD14 and TLR4 between HC and PFC astrocytes at DIV17. N = 6 independent astrocyte cultures. **D** Representative western blots showing protein expression levels of Aldh1L1, GFAP, CD14 and TLR4 relative to actin expression in cultured HC and PFC astrocytes

Here, we questioned whether the response to LPS would vary between astrocytes isolated from the hippocampus (HC) and from the prefrontal cortex (PFC), as these brain regions are particularly vulnerable to neuroinflammatory perturbations. We therefore cultured primary HC and PFC astrocytes isolated from the same neonatal mice to have matching conditions (Fig. 1A). On day in vitro (DIV) 11, astrocytes were infected with AAV-hGFAP-tdTomato-V5-GCaMP6s, a ratiometric Ca^2+^ biosensor to visualize cell morphology and report Ca^2+^ activity. Ca^2+^ imaging was conducted between DIV14-17 before fixation and further analysis. Importantly, serum exposure during cultivation has been shown to cause astrocyte reactivity, resulting in fibroblast-like morphology and transcriptomic changes (Zhang et al. 2016; Perriot et al. 2018). Therefore, we used serum-free protocols for both, the cultivation of mouse primary astrocytes and the human iPSC-derived astrocytes to acquire quiescent astrocytes for this study. Microscopy analysis revealed that at DIV17, astrocytes from both the HC and PFC show expression of the glial fibrillary acidic protein (GFAP), as well as the LPS receptors Cluster-of-differentiation (CD)14 and Toll-like receptor 4 (TLR4; Fig. 1B). The two-dimensional culture configuration results in substantial spatial overlap of immunolabeling patterns; nevertheless, higher-magnification inspection (not shown) reveals distinct differences in the fine-scale structural distribution of CD14, TLR4, and GFAP. RNA isolation and RT-qPCR revealed different expression profiles between HC and PFC astrocytes: As expected, PFC astrocytes expressed lower levels of mRNA encoding GFAP, while expression of mRNAs for both CD14 and TLR4 were elevated in PFC cultures (Fig. 1C). The expression of both receptors was further confirmed by western blot (Fig. 1D). Stimulation with 10 µg/ml LPS for 3 h did not change expression levels of both proteins (quantification not shown). To directly substantiate activation of an inflammatory program in our cultures, we have quantified the expression of canonical inflammatory cytokines (Suppl. Figure 1). We further audited the purity of our astrocyte cultures to exclude potential microglial contribution by analyzing the presence of microglia-specific Ionized calcium-binding adapter molecule 1 (Iba1; Suppl. Figure 2). In PFC astrocyte cultures, we detected 9 cells showing Iba1 immunofluorescence from a total of 4051 counted cells (0.22%). In HC astrocyte cultures, we found 2 Iba1^+^ cells from 1961 counted cells (0.10%), indicating near-complete absence of microglia. Thus, these mouse primary astrocyte cultures represent a suitable model for studying the impact of LPS-induced inflammation on astrocyte Ca^2+^ activity.

### LPS affects the Ca^2+^ activity of mouse HC and PFC astrocytes

**Fig. 2:**
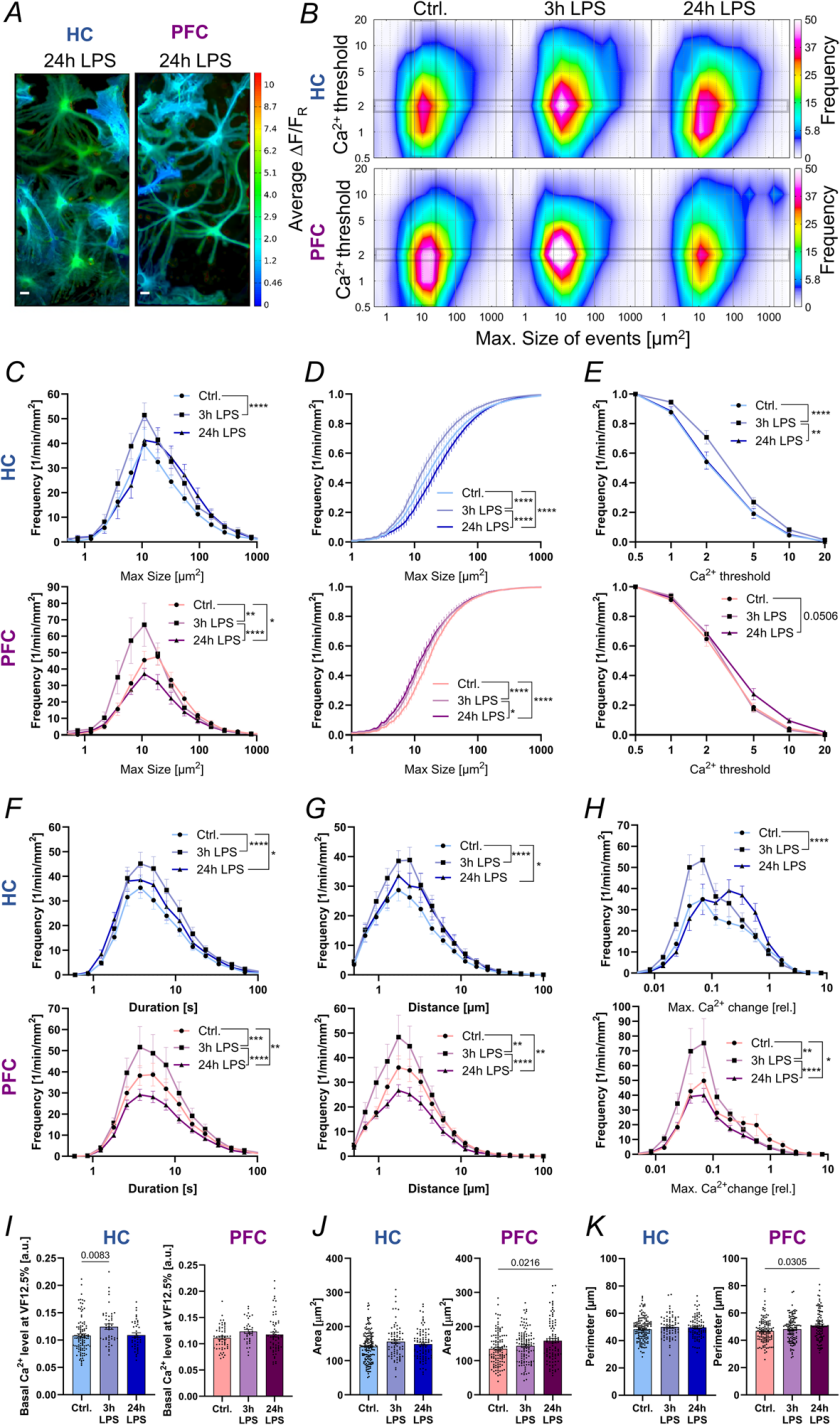
LPS stimulation affects the Ca^2+^ activity and morphology of mouse hippocampal and prefrontal cortex astrocytes. **A** Representative images of average ΔF/F_R_ of astrocytes from the mouse HC and PFC after 24 h of LPS treatment. Scale bars 20 µm. **B** Color-coded frequency distribution of maximum size of Ca^2+^ events across the Ca^2+^ thresholds 0.5—20 of mouse HC (top row) and PFC (bottom row) astrocytes. **C** Frequency distribution in HC astrocytes (top) and PFC astrocytes (bottom) of maximum size of Ca^2+^ events at Ca^2+^ threshold 2 for unstimulated (Ctrl.) and after 3 h and 24 h of LPS treatment. **D** Cumulative frequency of Ca^2+^ event size at Ca^2+^ threshold 2 and **E** across Ca^2+^ thresholds for Ctrl. astrocytes and after 3 h and 24 h of LPS treatment. **F** Frequency distributions at Ca^2+^ threshold 2 for Ca^2+^ event duration, **G** distance, and **H** maximum slope of Ca^2+^ change for HC (top row) and PFC astrocytes (bottom row). **I** Basal Ca^2+^ levels at volume fraction (VF) 12.5% for HC and PFC astrocytes in the Ctrl. condition and after 3 h and 24 h of LPS stimulation. **J** Area and **K** perimeter of astrocyte nuclei from the HC and PFC for all three conditions, respectively. Data in B-H from HC: *n* = 39,27,19 recordings and PFC: *n* = 19,29,20 recordings. Data in I correspond to HC: *n* = 105, 45, 41 cells and PFC: *n* = 54, 35, 62. Data in J-K from HC: *n* = 148, 75, 86 cells and PFC: *n* = 116, 116, 95 cells; all for Ctrl., 3 h, 24 h LPS, respectively, and from N ≥ 5 independent cultures

Ca^2+^ activity in astrocytes can be regarded as an indicator of their arousal state, even though the exact consequences of altered Ca^2+^ activity are not yet known. The Ca^2+^ biosensor AAV-hGFAP-tdTomato-V5-GCaMP6s was used in a ratiometric approach to assess Ca^2+^ signaling in cultured astrocytes at DIV14 under basal conditions (Ctrl.) as well as after 3 h and 24 h LPS exposure (Fig. 2A, Suppl. Figure 3, Suppl. Videos SV1-3). In all investigated conditions, endogenous Ca^2+^ activity was detected as ΔF/F_R_ and analyzed using our multi-threshold event detection (MTED) algorithm to define maximum event sizes, durations, distance propagation and maximum slope of Ca^2+^ change (Müller et al. 2021), (see Suppl. Table T1). Figure 2B depicts the distribution of Ca^2+^ event sizes for HC astrocytes (top row) and PFC astrocytes (bottom row) in unstimulated (Ctrl.) cells and after 3 h and 24 h of LPS exposure. The Ca^2+^ thresholds on the y-axis represent specific levels of Ca^2+^ as $\Delta F/{F}_{R}$, while the frequency of Ca^2+^ event occurrence is color-coded. The highest occurrence of Ca^2+^ events were detected at Ca^2+^ threshold 2, reflecting events in which the amount of Ca^2+^ almost increased by factor 2, and were therefore further analyzed. Both HC and PFC astrocytes exhibited a significantly higher frequency of Ca^2+^ events after 3 h LPS stimulation (Fig. 2C, HC + 35%, PFC + 28%), while these events were smaller in size, as visible by the left-shift of the corresponding curve shown in Fig. 2D (HC −24%, PFC −17%). In HC astrocytes, the Ca^2+^ events after 3 h of LPS treatment reached higher Ca^2+^ thresholds (Fig. 2B, E; Ca^2+^ threshold 5: + 30%; threshold 10: + 100%), meaning they reached higher levels of cytosolic Ca^2+^. Interestingly, PFC astrocytes showed a different response than HC astrocytes to 24 h LPS stimulation, presenting reduced Ca^2+^ event size and frequency. Of note, some rare but very large Ca^2+^ events were detected, as can be seen from the 24 h LPS plot in Fig. 2B bottom row, and the Ca^2+^ events shifted to greater Ca^2+^ thresholds (Fig. 2E).

We further analyzed the Ca^2+^ event duration, distance and maximum slope of Ca^2+^ change, and found that HC astrocytes showed increased frequency after LPS stimulation, while PFC astrocytes presented increased frequency after 3 h LPS stimulation but significantly reduced the frequency after 24 h of LPS stimulation (Fig. 2F).

Scaling the GCaMP6s Ca^2+^ indicator signal to the expression of the covalently linked tdTomato intensity, enables to cancel out the indicator concentration and allows for the comparison of relative basal Ca^2+^ levels (Fig. 2I). We next applied volume fraction (VF) analysis (Medvedev et al. 2014; Minge et al. 2021) to compare basal Ca^2+^ levels in the astrocyte periphery (i.e., small VFs up to 12.5%), which are particularly important for the induction of Ca^2+^ events (Wu et al. 2019). Interestingly, we detected significantly elevated basal Ca^2+^ levels of about 10% in HC astrocytes 3 h after LPS stimulation, which could be a reason for higher Ca^2+^ threshold levels obtained in this condition (Fig. 2B, E). Noteworthy, basal Ca^2+^ levels were indifferent to control conditions after 24 h of LPS treatment, underlining that HC astrocytes show a rather acute and temporary restricted response to LPS. In contrast, in PFC astrocytes basal Ca^2+^ levels were not affected by LPS treatment.

However, we noticed that the nuclei of stimulated astrocytes appeared swollen, and analyzed the area (Fig. 2J) and perimeter (Fig. 2K) of the nuclei. Quantification revealed that this effect was only statistically significant in mouse PFC astrocytes after 24 h of LPS incubation (+ 20%), further indicating that HC and PFC astrocytes are differentially affected by LPS.

To control for LPS-induced apoptotic cell death, we investigated the activation of Caspase-3/7, which is a hallmark of apoptosis. Neither a reduction in cell numbers, nor an increase in Caspase3/7 activity after 24 h of treatment with 100 ng/ml LPS was observed (Suppl. Figure 4). These results demonstrate that the measured changes in nuclear morphology and Ca^2^⁺ signaling are not associated with cytotoxicity or apoptotic cell death, supporting the interpretation that the LPS-induced effects reflect viable, adaptive astrocytic responses rather than degenerative artifacts. Together with the overall decreased Ca^2+^ activity in PFC astrocytes 24 h after LPS stimulation, these findings suggest that PFC astrocytes might be more vulnerable to inflammation. 

### Human iPSC-derived astrocytes express GFAP, CD14 and TLR4

**Fig. 3:**
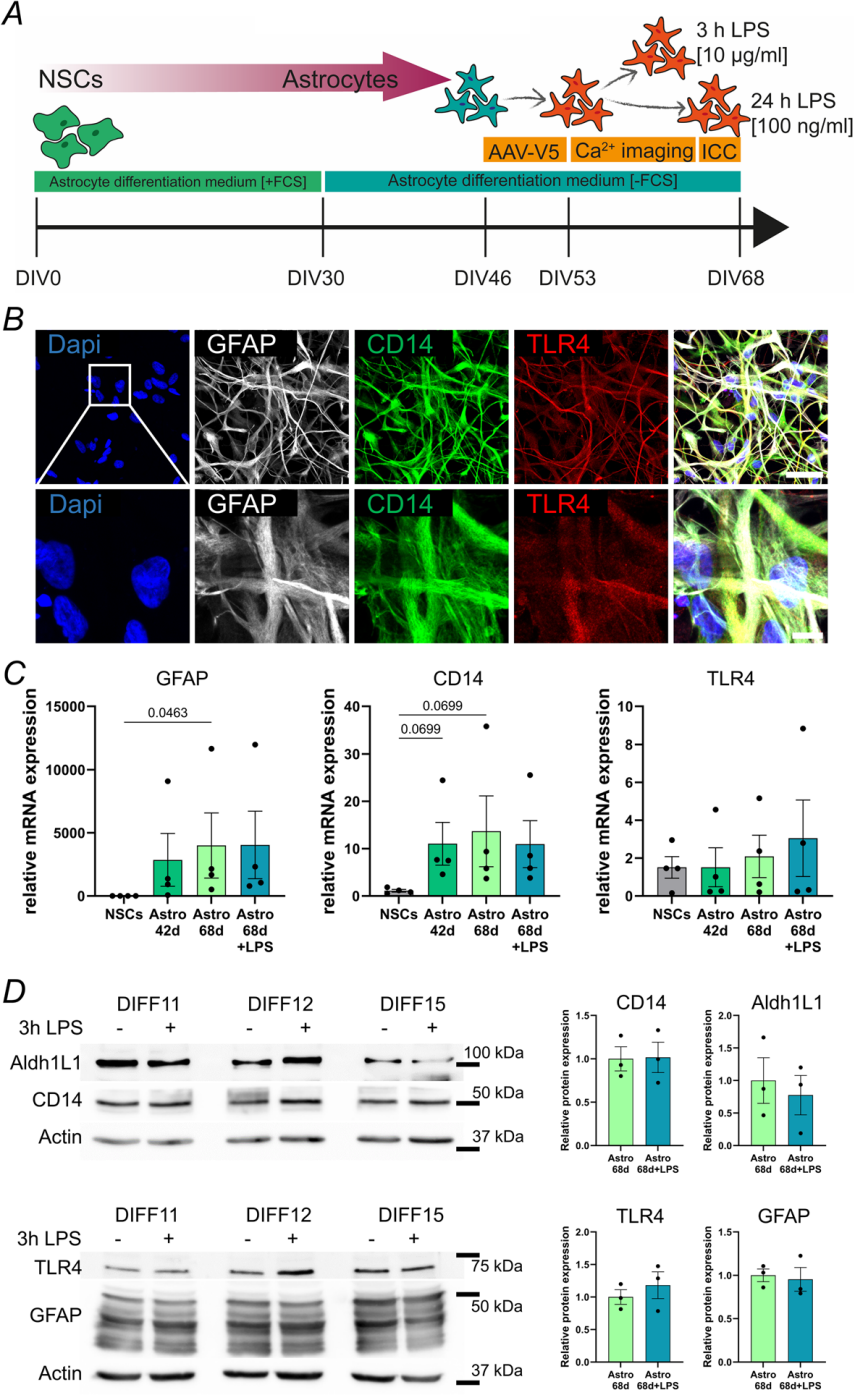
Human iPSC-derived astrocytes express GFAP, CD14 and TLR4. **A** Scheme of differentiation of neural stem cells (NSC) into astrocytes and experimental timeline. At DIV46, astrocytes were infected with Ca^2+^ biosensor AAV-hGFAP-tdTomato-V5-GCaMP6s. Ca^2+^ imaging was conducted after DIV53 without stimulation or after 3 h and 24 h of LPS treatment. **B** Immunocytochemistry demonstrates expression of GFAP, CD14 and TLR4 in differentiated hiPSC-derived astrocytes at DIV68. Scale bars 50 µm (top) and 10 µm (magnification). **C** Relative mRNA expression levels of GFAP, CD14 and TLR4 during different stages of the differentiation and after 3 h of LPS stimulation (10 µg/ml) at DIV68, *N* = 4 independent astrocyte differentiations. **D** Representative western blots and quantification showing GFAP, Aldh1L1, CD14 and TLR4 expression levels at DIV68 before and after LPS stimulation (3 h, 10 µg/ml), *N* = 3 independent astrocyte differentiations

Human astrocytes are known to be more complex than rodent astrocytes in regard to their morphology and function, and differently respond to inflammation (Zhang et al. 2016). The investigation of human cells has become more feasible due to recent progress in the directed differentiation of neural stem cells (NSCs) into specific neural cell types, enabling controlled studies of human cellular function and development. In the present study, we generated human induced pluripotent stem cell (hiPSC)-derived astrocytes to investigate their endogenous Ca^2+^ activity and compare their response to LPS exposure with mouse astrocytes. Figure 3A depicts the experimental timeline of hiPSC-derived astrocyte differentiation and experiments. At DIV30, we induced a stellate morphology by changing to serum-free culturing medium, followed by infection with AAV-hGFAP-tdTomato-V5-GCaMP6s at DIV46. LPS treatment and subsequent Ca^2+^ imaging were conducted starting at DIV53. At DIV68, astrocytes were fixed for immunocytochemistry, or lysed for western blot and mRNA isolation. Proper differentiation to astrocytes was confirmed by expression of specific cellular markers (Suppl. Figure 5–6). Immunocytochemistry showed expression of astrocytic markers GFAP (90 ± 8.5% GFAP^+^ cells), Aldh1L1 (93 ± 11.6% Aldh1L1^+^ cells) and S100b (94 ± 5.1% S100b^+^ cells) at DIV68.

Using confocal microscopy, we demonstrated the expression of LPS receptors CD14 and TLR4 in our hiPSC-derived astrocytes at DIV68. As shown in Fig. 3B, both LPS receptors co-localized with GFAP. In addition, we assessed the mRNA expression levels of GFAP, CD14 and TLR4 in the initial NSCs, at DIV42 and at DIV68 without and with 3 h of LPS treatment (Fig. 3C). GFAP mRNA expression levels are rising significantly throughout the differentiation process, while mRNA encoding CD14 is strongly increased in differentiated astrocytes compared to NSCs. Of note, expression of TLR4 mRNA was detectable in both NSCs and hiPSC-derived astrocytes. Interestingly, 3 h LPS treatment at DIV68 did not significantly affect the relative expression, neither on mRNA (Fig. 3C) or protein level (Fig. 3D). Overall, hiPSC-derived astrocytes express CD14 and TLR4 and are therefore a suitable model to investigate the LPS-induced inflammatory influences on human astrocyte Ca^2+^ activity.

### Human iPSC-derived astrocytes change their Ca^2+^ activity in response to LPS stimulation

**Fig. 4:**
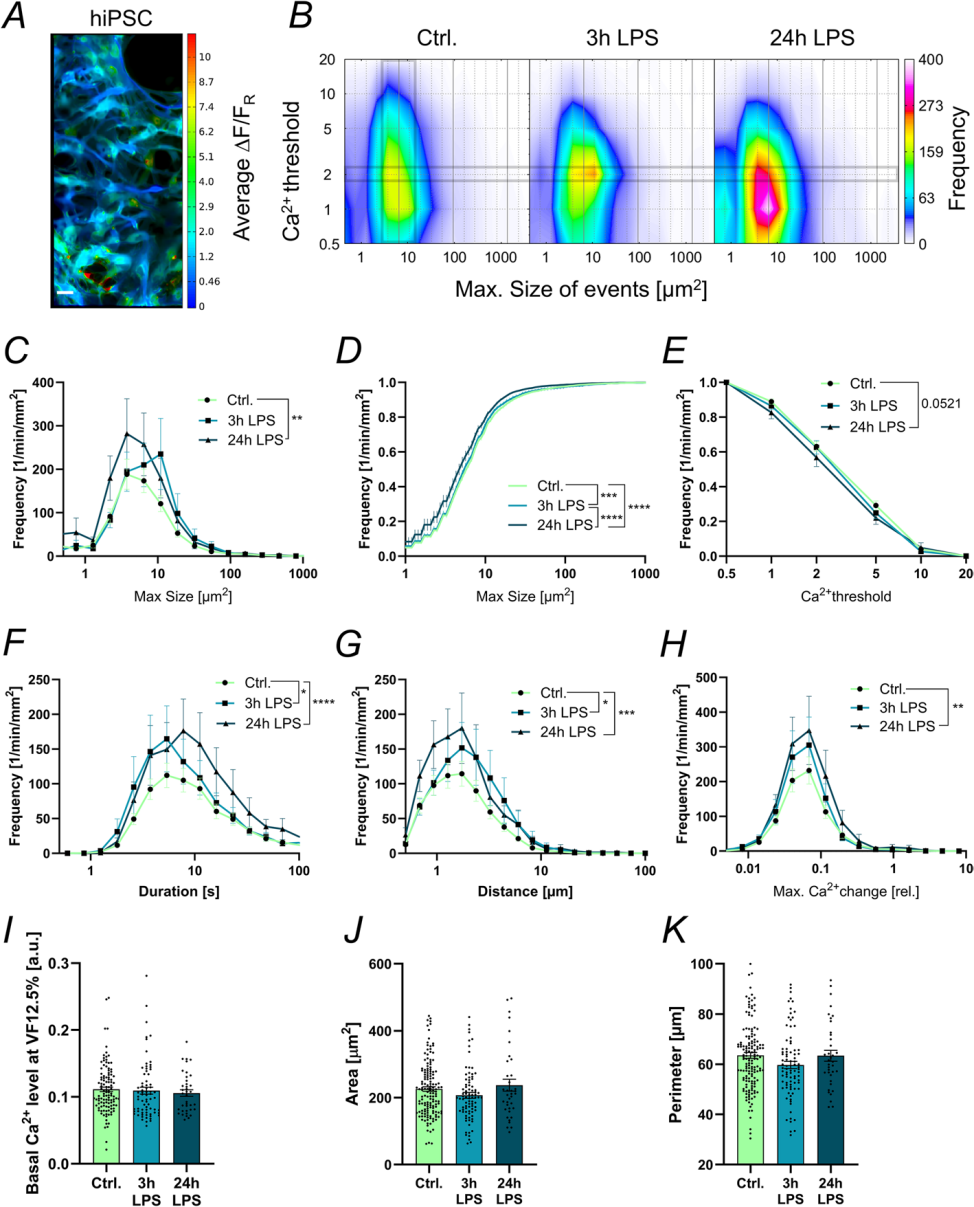
LPS stimulation affects the Ca^2+^ activity of hiPSC-derived astrocytes. **A** Representative image of average ΔF/F_R_ of hiPSC-derived astrocytes. Scale bar 20 µm. **B** Color-coded frequency distribution of maximum size of Ca^2+^ events across the Ca^2+^ thresholds 0.5—20 in hiPSC-derived astrocytes. **C** Frequency distribution of maximum size of Ca^2+^ events at Ca^2+^ threshold 2 for unstimulated (Ctrl.) hiPSC-derived astrocytes and after 3 h and 24 h of LPS treatment. **D** Cumulative frequency of Ca^2+^ event size at Ca^2+^ threshold 2 and **E** across Ca^2+^ thresholds for Ctrl. astrocytes and after 3 h and 24 h of LPS treatment. **F** Frequency distributions at Ca^2+^ threshold 2 for Ca^2+^ event duration, **G** distance, and **H** maximum slope of Ca^2+^ change for hiPSC-derived astrocytes. **I** Basal Ca^2+^ levels at volume fraction (VF) 12.5% in hiPSC-derived astrocytes in the Ctrl. condition and after 3 h and 24 h of LPS stimulation. **J** Area and **K** perimeter of hiPSC-derived astrocyte nuclei for all three conditions, respectively. Data in B-H *n* = 186, 104, 56 recordings. Data in I correspond to *n* = 114, 72, 38 cells. Data in J-K from *n* = 154, 86, 37 cells; all for Ctrl., 3 h, 24 h LPS, respectively, and from N ≥ 6 independent differentiations

Having demonstrated that the hiPSC-derived astrocytes express receptors for LPS, we treated the Ca^2+^-biosensor-expressing cells according to the standard LPS protocol at DIV53, either with 10 µg/ml LPS for 3 h or 100 ng/ml LPS for 24 h. We then recorded their Ca^2+^ activity and performed MTED analysis (Fig. 4A). The hiPSC-derived astrocytes displayed a very high occurrence of Ca^2+^ events under basal (unstimulated) conditions (Fig. 4B), which was about 5- to 8-fold higher in frequency than in cultured mouse astrocytes (compare Fig. 2B). The majority of these events, however, reached only the lower Ca^2+^ thresholds 1 and 2 (Fig. 4B). The distribution of Ca^2+^ events at Ca^2+^ threshold 2 was further analysed to investigate the influence of LPS treatment on endogenous Ca^2+^ activity of hiPSC-derived astrocytes. Figure 4C-H shows that Ca^2+^ event frequency is greatly enhanced after 3 h (+ 30%) and especially after 24 h of LPS treatment (+ 60%). The latter events were, however, smaller (as indicated by the left shift of the curve in Fig. 4D) and incorporated less Ca^2+^ (Fig. 4E). Of note, the basal Ca^2+^ levels (Fig. 4I), as well as nuclei size and perimeter were not affected by LPS treatment (Fig. 4J-K).

Comparing human with mouse astrocytes, hiPSC-derived astrocytes show a ~ 3 times smaller Ca^2+^ event size, which goes along with a 50% longer event duration (compare Fig. 2C and F–H with 4 C and F–H, Suppl. Figure 7). However, the slope of Ca^2+^ change is about one order of magnitude smaller in hiPSC-derived astrocytes for Ca^2+^ thresholds greater 2 (Suppl. Figure 7). Taking together, the 5- to 8-fold increased frequency of Ca^2+^ events observed in hiPSC-derived astrocytes must be assumed to result from faster Ca^2+^ reuptake into the endoplasmic reticulum rather than being the result of slower Ca^2+^ release.

### Comparison of mouse and human astrocytes’ response to LPS

**Fig. 5:**
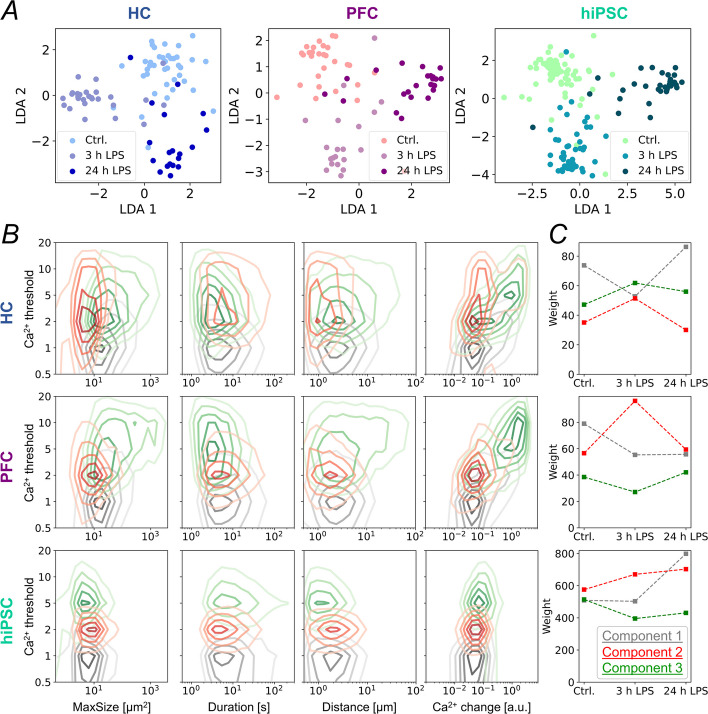
Dissection and comparison of mouse and human astrocyte response to LPS. **A** Linear Discriminant Analysis (LDA) was applied to HC, PFC and hiPSC astrocyte Ca^2+^ datasets. **B** Non-Negative Matrix Factorization (NMF) yielded in 3 components incorporating characteristic features of Ca^2+^ event size, duration, distance, and maximum slope of Ca^2+^ change. **C** Weights of the NMF separation, visualized for all three components without LPS stimulation (Ctrl.), after 3 h and after 24 h LPS treatment

Astrocytes represent a heterogeneous cell population, characterized by distinct molecular profiles and functional specializations. To resolve subpopulations within our datasets (i.e., stimulus responders versus non-responders) we employed computational clustering methods and component analysis to identify and classify distinct astrocytic response patterns (Fig. 5, Suppl. Figure 8). Linear Discriminant Analysis (LDA) was applied to compare not only individual but a combination of relevant Ca^2^⁺ parameters, including event size, duration, spatial distance spread, and the slope of Ca^2^⁺ transients. This approach enabled successful separation between astrocyte recordings under control conditions (Ctrl.) and those after 3 h and 24 h of LPS treatment (Fig. 5A). These separations were visible for all three datasets, from HC, PFC and hiPSC-derived astrocytes, indicating that there are distinct patterns of LPS-induced changes in astrocyte Ca^2+^ activity in all three investigated astrocyte populations. The partial overlap between groups might be attributed to not all cells in the field of view showing a measurable response to LPS.

We furthermore assume that the observed pattern of Ca^2+^ activity might be a non-negative linear combination of the Ca^2+^ activity of responding and non-responding cells. We therefore applied Non-Negative Matrix Factorization (NMF) to retrieve a decomposition of individual components to identify the underlying characteristics. Here we treated all four previously investigated Ca^2+^ parameters (X) as superposition of three components (H) (Fig. 5B) and their weights (W) for each recording (Fig. 5C). As we already know that HC, PFC and hiPSC show different responses to LPS treatment, we applied NMF to the groups separately. In all three NMF results we found (i) a component characterized by small events with minimal Ca^2+^ changes (grey colour in Fig. 5B), (ii) a component incorporating moderate amount of Ca^2+^ elevation with smaller, long-lasting, and less propagating events (red colour), and (iii) a third component representing events which reach the highest Ca^2+^ thresholds (green colour). Interestingly, NMF revealed the same correlation between maximal event size and event duration (in the second and third component; red and green colour) as observed comparing Ca^2+^ event characteristics between mouse and hiPSC-derived astrocytes: smaller event size correlates with longer duration, and larger size with shorter duration. We also tested NMF using 2 to 10 components, acknowledging that the reconstruction error decreases with the increasing number of components. However, there is no clear evidence for an optimal number of components. Due to the increasing complexity with an increasing number of components, we decided to present NMF results based on three components.

Figure 5C shows the mean of the weights of the NMF separation, visualized for all three components without LPS stimulation (Ctrl.), after 3 h and after 24 h LPS treatment. In HC astrocytes, the red and green components account for the observed increase in event frequency after 3 h of LPS treatment, accompanied by a reduction in the grey component. After 24 h of LPS treatment, the component distribution resembles that of the control condition (Fig. 5C top). In PFC and hiPSC-derived astrocytes, the red component of events is responsible for the increase in overall Ca^2+^ activity after 3 h of LPS treatment. However, in PFC astrocytes the component distribution after 24 h of LPS treatment is similar to that of the control condition, whereas in hiPSC-derived astrocytes, the contribution of all three components is higher compared to the 3 h treatment group (Fig. 5C bottom).

Taken together, the Ca^2+^ activity patterns of all three astrocyte populations are differentially affected by LPS stimulation. While in HC astrocytes medium and large events (green and red components, Fig. 5) increase after 3 h of LPS treatment, they are replaced by predominantly small Ca^2+^ events (grey component, Fig. 5) following 24 h LPS exposure. In PFC astrocytes, medium events increase 3 h after LPS treatment, while a markable reduction in small events is observed after 24 h. In contrast, hiPSC-derived astrocytes exhibit increased small and medium Ca^2+^ events after both 3 h and 24 h LPS treatment.

## Discussion

Our study differs substantially from previous investigations of LPS effects on astrocyte Ca^2+^ activity by examining endogenous astrocyte activity in absence of exogenous triggering stimuli. Within, we have dissected the endogenous Ca^2+^ activity of mouse primary culture and human iPSC-derived astrocytes after exposure to LPS in a comparative approach. The key findings are that i) cultured mouse and human astrocytes express LPS receptors CD14 and TLR4, and show increased Ca^2+^ event frequency after 3 h exposure to LPS, ii) mouse PFC astrocytes show reduced Ca^2+^ activity after 24 h of LPS treatment accompanied by enlarged nuclei, iii) mouse HC and hiPSC-derived astrocytes exhibit an increased frequency of low-Ca^2^⁺-threshold events after 24 h of LPS stimulation, iv) hiPSC-derived astrocytes show a distinctly different Ca^2+^ activity pattern compared to mouse primary astrocytes, even under unstimulated control conditions. Concluding, the effect of LPS on endogenous Ca^2+^ activity is different between mouse HC and PFC astrocytes and between mouse and human astrocytes, highlighting the regional diversity of astrocytes across brain regions and species.

Primary cultures from mouse brain tissue are a widely used model to study different aspects of cellular signaling. It has been repeatedly shown, that fundamental features of brain cells are preserved under appropriate culturing conditions (Lange et al. 2012), including astrocytic Ca^2+^ activity (Wu et al. 2014).

For the translation of research findings acquired in mouse primary cultures to human conditions, a first crucial step is to validate the results obtained in mouse model cells in human primary cells. Despite continuous advancement in the last decade, the cultivation conditions of human iPSC-derived cells, especially astrocytes, are not yet standardized, leading to very different states of differentiated cells between protocols (reviewed in Lanciotti et al. (2021)). It is therefore not certain, that results obtained from our hiPSC-derived astrocytes would be identical across different culturing protocols. Additionally, this study relied on hiPSC-derived astrocytes from a single donor, and donor-specific factors such as sex and age may influence the generalizability of the results. Careful monitoring and characterization of differentiated cells remain necessary, until more standardized culturing conditions are likely to be achieved in the coming years.

LPS-induced inflammation is one of the most widely-used and well-characterized models to study inflammation-associated signalling. Among others, LPS treatment induces key inflammatory hallmarks, such as cytokine storms of IL-1, IL-6, TNF-α and CXCL12 (see also Suppl. Material 1). Advantages of this model include low costs, reproducibility, as well as in vitro and in vivo application (Sheen et al. 2024). It was further found to be most suited for studying acute inflammatory signalling in vivo (Seemann et al. 2017). Even though LPS was mostly shown to not cross the blood–brain-barrier in vivo, the systemic inflammatory reaction leads to neuroinflammation, cognitive impairment and behavioural deficits similar to the various symptoms of neurodegenerative disorders. Therefore, LPS can also be used in models to mimic neurodegenerative diseases (Zhao et al. 2019; Batista et al. 2019; Skrzypczak-Wiercioch and Sałat 2022). High dose LPS treatment in vivo (3 mg/kg in mice) can lead to brain-blood-barrier disruption within 24 h, among others in the brain regions HC and frontal cortex (Banks et al. 2015).

In vitro, the expression of LPS-receptors CD14 and TLR4 in astrocytes was previously described, and the individual response of mouse and human astrocytes to LPS stimulation was compared by analysing cytokine production (Tarassishin et al. 2014). The authors of this study further concluded that CD14 is not needed for the LPS-induced inflammatory response in mouse astrocytes, and that human foetal astrocytes do not respond themselves, but that microglia are needed to produce a first response, which in turn then activates astrocytes. They hypothesize, that in past studies reporting on astrocyte responses to LPS, microglial contamination in the cultures might have induced these effects. In our study, however, we used hiPSC-derived astrocytes generated from NSCs, which can be the origin of neuronal cells and astrocytes, but not microglia. Microglial cells originate from the yolk sac, and astrocytes are of ectodermal origin (Verkhratsky and Nedergaard 2018; Saijo and Glass 2011). Therefore, microglial signalling cannot account for the LPS-induced effects on Ca^2+^ activity in our hiPSC-derived astrocytes.

In our study we focused on two well-accepted LPS-stimulation protocols: a high-dose treatment for 3 h and low-dose exposure for 24 h (Tarassishin et al. 2014; Shemi et al. 2000). Notably, the endogenous Ca^2+^ activity of mouse HC astrocytes after 24 h of LPS treatment was similar to control conditions, while marked differences were observed in PFC astrocytes, which showed reduced activity, and in hiPSC-derived astrocytes, which exhibited increased activity. These findings highlight the distinct responsiveness of different astrocyte populations and suggest promising directions for future research. Notably, different astrocyte populations may express distinct levels of certain LPS receptors, which can shape the consequences of LPS stimulation for astrocyte Ca^2+^ activity. Further studies should investigate the temporal progression of astrocyte Ca^2+^ profiles following LPS exposure, identify when peak activity occurs, and clarify whether prolonged LPS treatment leads to sustained adaptations or a recovery of astrocyte function.

Strokin and colleagues applied purinergic ATP stimulation after LPS treatment and found significantly increased amplitudes and durations of astrocyte Ca^2+^ events. These changes were accompanied by increased Ca^2+^ load in the ER, and were linked to the expression and activity of VIA phospholipase A2 (Strokin et al. 2011). Whether the ER Ca^2+^ load in our three different astrocyte preparations is similarly affected, or basal ER Ca^2^⁺ levels differ between them, remain important future questions as chronic alteration of astrocyte Ca^2+^ activity is considered as a potential driver of inflammation-associated neurodegenerative disorders, with certain brain areas appearing more vulnerable than others.

Aberrant Ca^2+^ activity of astrocytes has been reported in numerous brain diseases. For example, reduced Ca^2+^ signalling has been observed after status epilepticus and in a mouse model of Huntington’s disease (Jiang et al. 2016; Plata et al. 2018). Regional differences have also been reported, with astrocytes from the hippocampus and entorhinal cortex showing distinct alterations in Ca^2^⁺ dynamics in a mouse model of AD (Grolla et al. 2013). It remains however to be clarified, whether aberrant astrocyte Ca^2+^ activity is an initiator, driver or secondary consequence of these pathological processes.

Astrocyte reactivity in response to inflammation was shown to be dependent on the Ca^2+^ channel Orai1. Inhibition of Orai1 ameliorated inflammation-associated behavioural changes in mice (Novakovic et al. 2023). Birla et al*.* further identified Orai1 as a primary effector of TLR4 signalling, acting as a Ca^2+^ leak channel, maintaining astrocytic Ca^2+^ homeostasis in spinal cord astrocytes (Birla et al. 2022). Consistent with our findings, they reported that LPS stimulation via TLR4 signalling and upregulation of Orai1 expression elevates basal cytosolic Ca^2+^ levels in spinal astrocytes after 3 h of LPS exposure. We observed a similar elevation in HC but not in PFC astrocytes, further supporting the regional heterogeneity of astrocyte populations, and underlining the need to investigate distinct astrocyte subtypes individually.

Utilizing MTED, we systematically dissected the complexity of astrocyte Ca^2+^ activity patterns, integrating machine learning (ML) tools for a comprehensive analysis of underlying dynamics. ML approaches, particularly dimension reduction strategies, have been successfully used in prior studies of astrocyte Ca^2^⁺ signalling. For instance, principal component analysis (PCA) and independent component analysis (ICA) have been employed to classify signals and differentiate astrocyte activity from neuronal contamination (Mukamel et al. 2009; Weiser et al. 2023). In addition, t-distributed stochastic neighbour embedding (t-SNE) has been proposed for multivariate analysis of heterogeneous parameters for cluster analysis (Wang et al. 2019). While we evaluated PCA, ICA and t-SNE, we identified NMF as the optimal method for our data-set. Due to the non-negative nature of our frequency distributions and their linear dependence, NMF was best at detecting the underlying feature characteristics.

Our analysis revealed that hiPSC-derived astrocytes exhibited a five- to eight-fold increase in Ca^2+^ event frequency compared to mouse astrocytes, coupled with smaller maximum event sizes and prolonged event durations. In mouse astrocytes, NMF further demonstrated that smaller Ca^2+^ events were associated with extended durations. Event frequencies were quantified as spatial–temporal occurrences (events per unit time and space). Intriguingly, the elevated frequency observed in hiPSC-derived astrocytes arose from spatially distinct small events rather than temporal clustering. This spatial distinction suggests that hiPSC-derived astrocytes exhibit a higher density of localized active regions, driving their increased Ca^2+^ activity frequency relative to their murine counterparts. These findings underscore the utility of NMF in resolving spatially and temporally heterogeneous Ca^2+^ dynamics, offering novel insights into model-specific astrocyte behaviour.

Given the established role of astrocyte Ca^2^⁺ signaling in modulating neuronal excitability and synaptic transmission, the increased frequency and spatial distribution of Ca^2^⁺ events in hiPSC-derived astrocytes may reflect enhanced ability for localized regulation of neuronal circuits. This suggests that human astrocytes, through their dense and spatially heterogeneous Ca^2^⁺ signaling, could exert more nuanced control over synaptic activity and therewith shape network homeostasis—an essential consideration when modeling explicitly human neuronal circuit function and dysfunction using iPSC-based systems. Therefore, future studies will be essential to characterize Ca^2+^ signalling in healthy astrocytes from different brain regions, to assess how it is altered in disease conditions and connect this to changes in neuronal network function. A major challenge will be the generation of astrocyte models that accurately recapitulate brain-region specific features of human astrocytes.

## Conclusions

Overall, our findings demonstrate that distinct subsets of astrocytes respond differently to LPS-induced inflammation, highlighting astrocyte heterogeneity and indicating brain region-specific susceptibility to inflammatory stimuli. These differences are of particular relevance in the context of neuronal connectomics, where regionally and functionally distinct astrocyte populations may differentially modulate neuronal circuit integrity under inflammatory stress. A better understanding of the cellular properties that confer vulnerability may thus serve as a basis for developing strategies to preserve astrocyte function and maintain network homeostasis in human brain models.

## Supplementary Information

Supplementary Material 1: Supplementary Figure 1: LPS induces an inflammatory response in cultured astrocytes. mRNA expression levels of A IL-1β, B IL-6 and C TNF-α under control conditions and after 3 h or 24 h of LPS stimulation in mouse astrocytes from the HC and PFC, and hiPSC-derived astrocytes at d68 in vitro. D Quantification of TNF-α levels in the supernatant of hiPSC-derived astrocytes using Elisa assay.

Supplementary Material 2: Supplementary Figure 2: Immunofluorescence staining for microglia and astrocytes. A Immunohistochemistry shows clear distinction of GFAP-positive astrocytes and Iba1-expressing microglia in a mouse hippocampal brain slice. The same antibodies were used in B to detect microglia in primary cultures from the mouse HC and PFC. Scale bars 50 µm. C Percentage of Iba1^+^ cells in three independent astrocyte cultures from the mouse HC and PFC identified by immunocytochemistry. D Relative mRNA expression levels of GFAP and Iba1 in mouse HC and PFC cultures.

Supplementary Material 3: Supplementary Figure 3: Representative traces and Ca^2+^ analysis images of mouse and human iPSC-derived astrocytes. A Representative Ca^2+^ traces of mouse HC astrocytes without (Ctrl.), and after 3 h or 24 h of LPS stimulation showing ΔF/F_R_ changes. The areas from which the traces originate are shown in the color-coded maximum ΔF/F_R_ images below, respectively, with the color of each square matching the color of a respective trace above. The white contours enclose active areas. The middle image shows the average ΔF/F_R_, while the right images depict the fraction of time in which the cell regions were active, meaning they showed Ca^2+^ activity above threshold. The same is shown for B astrocytes from the mouse PFC and C human iPSC-derived astrocytes. Scale bars 20 µm.

Supplementary Material 4: Supplementary Figure 4: Influence of LPS treatment on apoptosis in astrocytes. Representative images of apoptosis assay in mouse A HC, C PFC astrocytes and E human iPSC-derived astrocytes. Cells were treated with 100 ng/ml LPS for 24 h. Scale bars 200 µm. Quantification of nuclei and caspase-TexasRed intensity relative to number of Hoechst-stained nuclei in B HC, D PFC, and F human iPSC-derived astrocytes.

Supplementary Material 5: Supplementary Figure 5: Human iPSC-derived astrocyte characterization during differentiation. Relative mRNA expression levels of Aldh1L1, Nestin, NeuN, Vimentin, SOX2 and S100b during different stages of the differentiation protocol and after 3 h of LPS stimulation (10 µg/ml) at DIV68.

Supplementary Material 6: Supplementary Figure 6: Human iPSC-derived astrocyte characterization by Immunocytochemistry. Stainings for A Aldh1L1, Nestin, B NeuN, Vimentin, C SOX2 and S100b at DIV68. Scale bars 50 µm (top) and 10 µm (bottom magnifications).

Supplementary Material 7: Supplementary Figure 7: Comparison of Ca^2+^ event characteristics between mouse and human iPSC-derived astrocytes. The color-coded frequency distribution of Ca^2+^ events across Ca^2+^ thresholds of mouse HC (top row), PFC (middle row) and human iPSC astrocytes (bottom row) is shown for various features. A Event duration, B Maximum distance the Ca^2+^ center moved, C The maximal temporal increase of Ca^2+^ (maximum slope of Ca^2+^ change).

Supplementary Material 8: Supplementary Figure 8: Individual component visualization from Non-negative Matrix Factorization. The three components of the feature characteristics H (event size, duration, distance, and maximum slope of Ca^2+^ change) from NMF for A HC, B PFC and C hiPSC-derived astrocytes. The color bar indicates the relative frequency of the components.

Supplementary Material 9: Supplementary Table1: Description of investigated Ca^2+^ parameters.

Supplementary Material 10: Supplementary videos 1-3: Representative Ca^2+^ activity and event detection in PFC astrocytes. Individual files show PFC astrocytes under unstimulated (Ctrl.) conditions (SV1), after 3 h LPS treatment (SV2) and after 24 h LPS treatment (SV3). The left videos show the processed GCaMP6s intensity. Middle videos show color-coded the calculated ∆F/F_R_ signal. Videos on the right show the detected events by the MTED algorithm.

## Data Availability

The data and MATLAB code developed in this study are available from the corresponding author upon request. The Python code is available on GitHub using the following link: https://github.com/kerstinlenk/QuantifyCalciumDynamics.

## Abbreviations

AD: Alzheimer’s disease
ALS: Amyotrophic lateral sclerosis
Ca^2+^: Calcium
CD14: Cluster-of-differentiation 14
CNS: Central nervous system
Ctrl: Control
DIV: Day in vitro
FTD: Frontotemporal dementia
GFAP: Glial fibrillary acidic protein
HC: Hippocampus
HD: Huntington’s disease
hiPSC: Human induced pluripotent stem cell
ICC: Immunocytochemistry
LDA: Linear Discriminant Analysis
LPS: Lipopolysaccharide
ML: Machine learning
MTED: Multi-threshold event detection
NMF: Non-Negative Matrix Factorization
NSC: Neural stem cells
PCA: Principal component analysis
PD: Parkinson’s disease
PFC: Prefrontal cortex
SEM: Standard error of the mean
TLR4: Toll-like receptor 4
t-sne: T-distributed stochastic neighbor embedding
